# Supplementary material for: DNA methylation at birth in monozygotic twins discordant for pediatric acute lymphoblastic leukemia
Source: Nat Commun. 2022 Oct 14;13:6077. doi: 10.1038/s41467-022-33677-z (PMC9568651; doi:10.1038/s41467-022-33677-z)
Supplement: Supplementary file 1 — Supplementary Information [file 41467_2022_33677_MOESM1_ESM.pdf]

Supplementary Figures

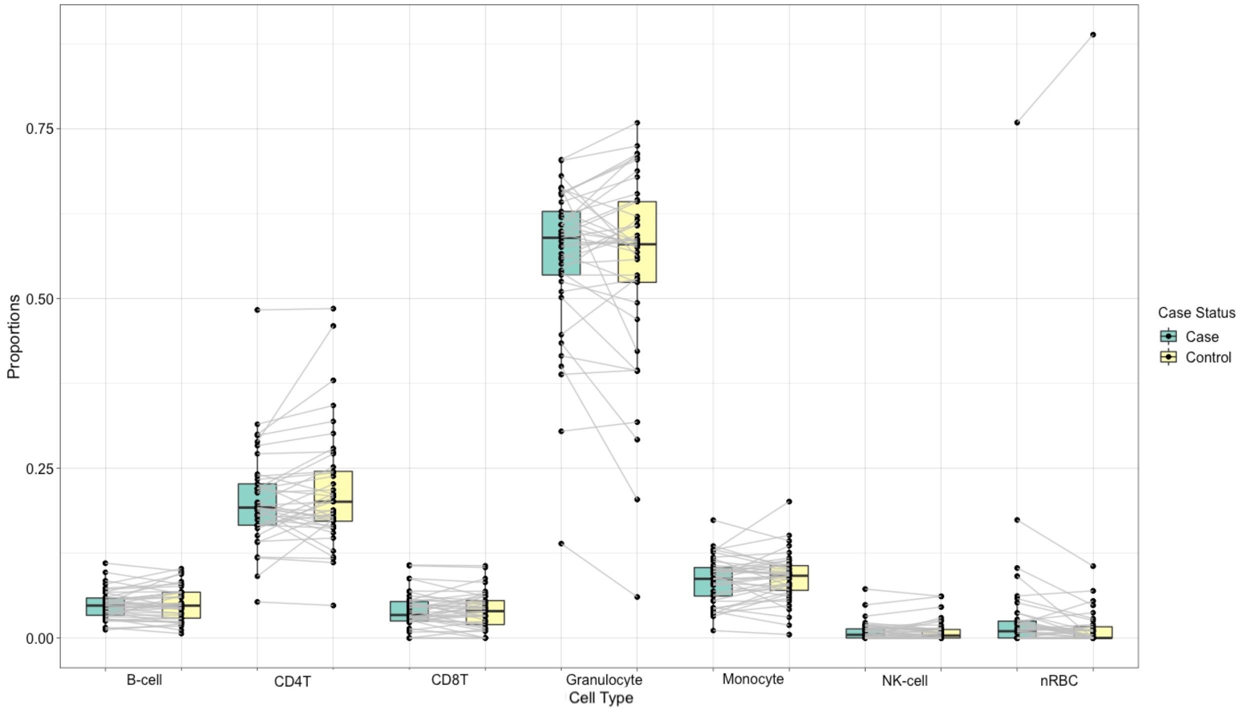

Supplementary Figure 1: Deconvolution analysis using IDOL method. Cell type composition by case status for *n* = 41 twin pairs is shown. Grey lines correspond to twin pairings. There were no significant differences in cell proportions by ALL case status for the 7 nucleated cell types assessed (two-sided paired Wilcoxon rank-sum). CD4T = CD4+ T-cell, CD8T = CD8+ T-cell, nRBC = nucleated red blood cell. Source data are provided as a Source data file. In boxplots, box represents interquartile range (IQR, first through third quartile) with centerline showing median value for all subjects, whiskers show minimum (first quartile minus 1.5*IQR) and maximum (third quartile plus 1.5*IQR) data range.

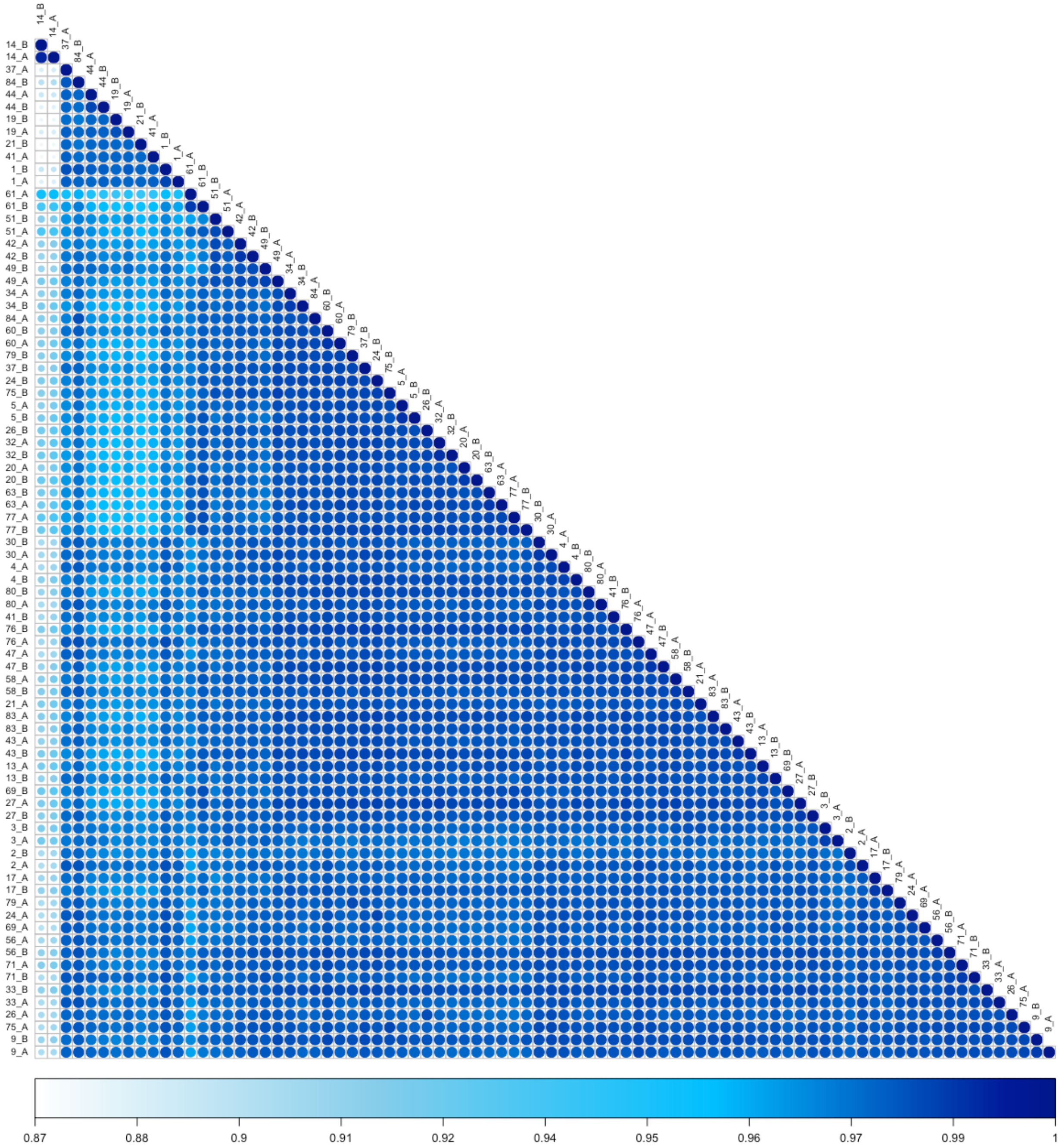

Supplementary Figure 2: Correlation matrix of DNA methylation beta values for all individuals in study. Correlation was calculated using the Spearman method for all individuals included in the study (*n* = 82) across all array probes (710,010 CpGs). Hierarchical clustering was applied for subject ordering on the plot. Source data are provided as a Source data file.

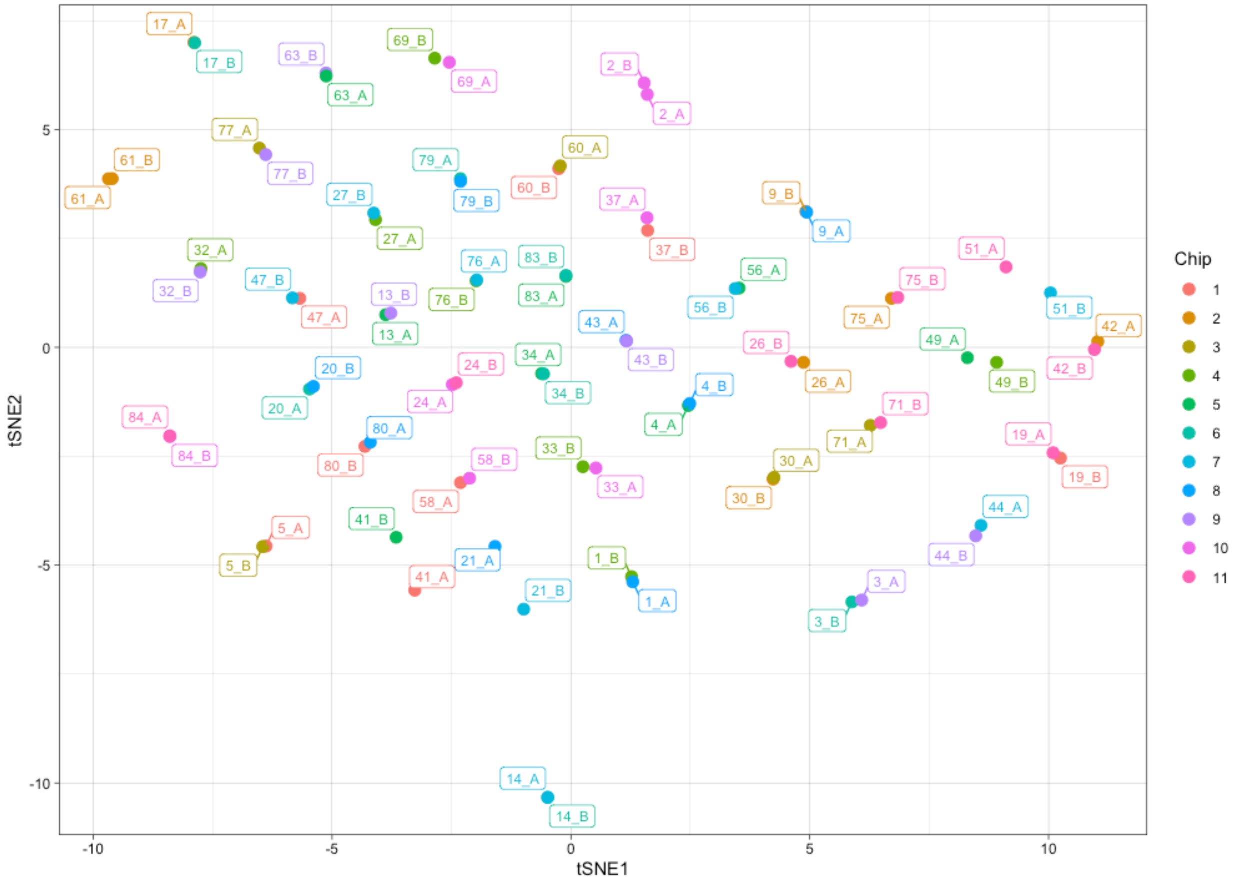

Supplementary Figure 3: tSNE analysis of DNA methylation data in all subjects. tSNE analysis was conducted on raw beta values for 695,997 CpG probes (omitting chromosomes X and Y) in all included subjects (*n* = 82 individuals) to evaluate for potential underlying technical artifact or systematic bias in the DNA methylation data structure. Plot labels correspond to individual twin pairs (twin A and twin B for each pair), colors correspond to array chip used in DNA methylation analysis. While twin pairing is tightly associated on the plot, the chip variable shows no obvious clustering pattern.

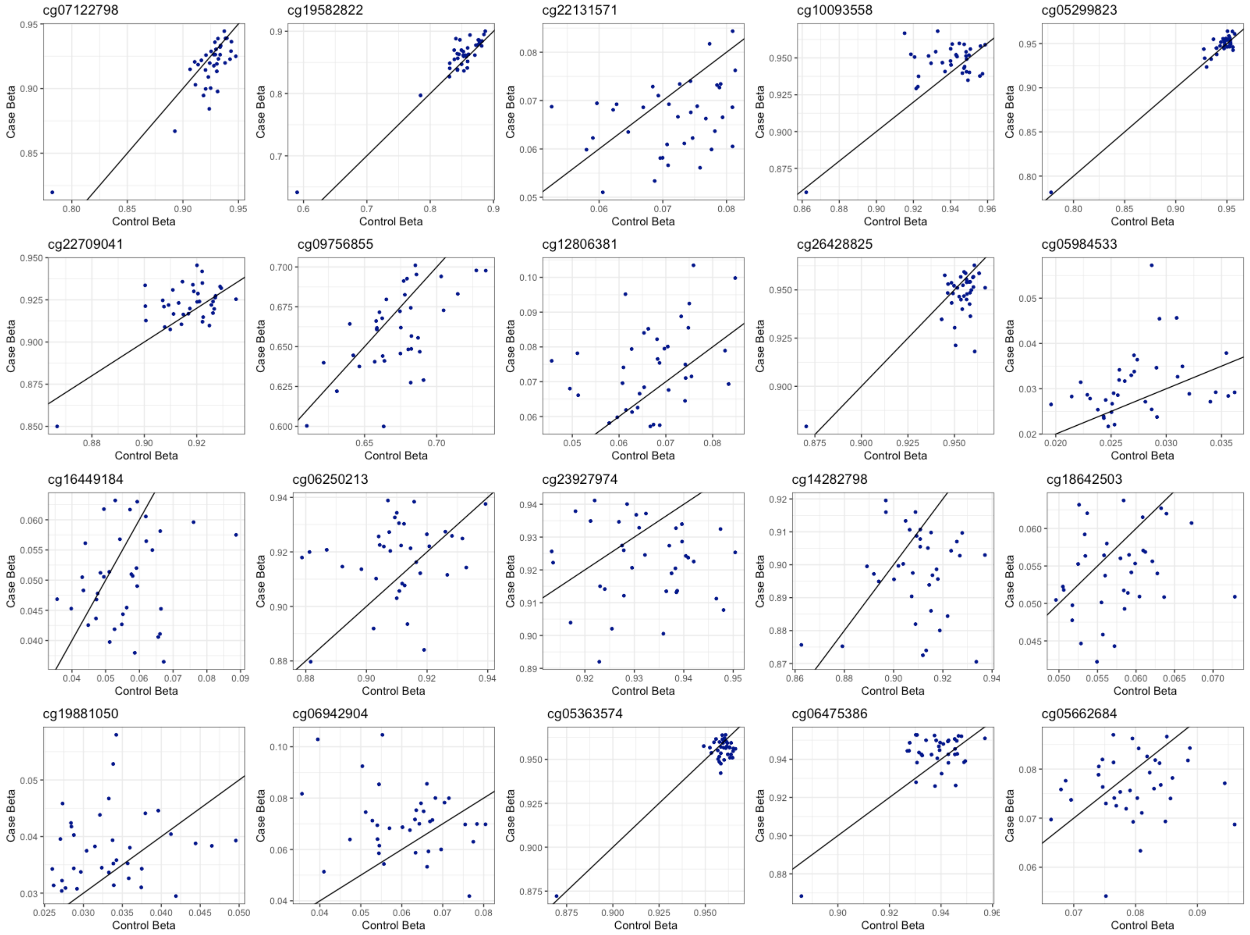

Supplementary Figure 4: Paired beta values in ALL cases and controls evaluated in conditional regression analysis (*n* = 37 pair) for the top 20 significant DMPs. Points above equivalence line (X = Y) indicate increased methylation in the case twin, while points below indicate decreased methylation in the case twin.

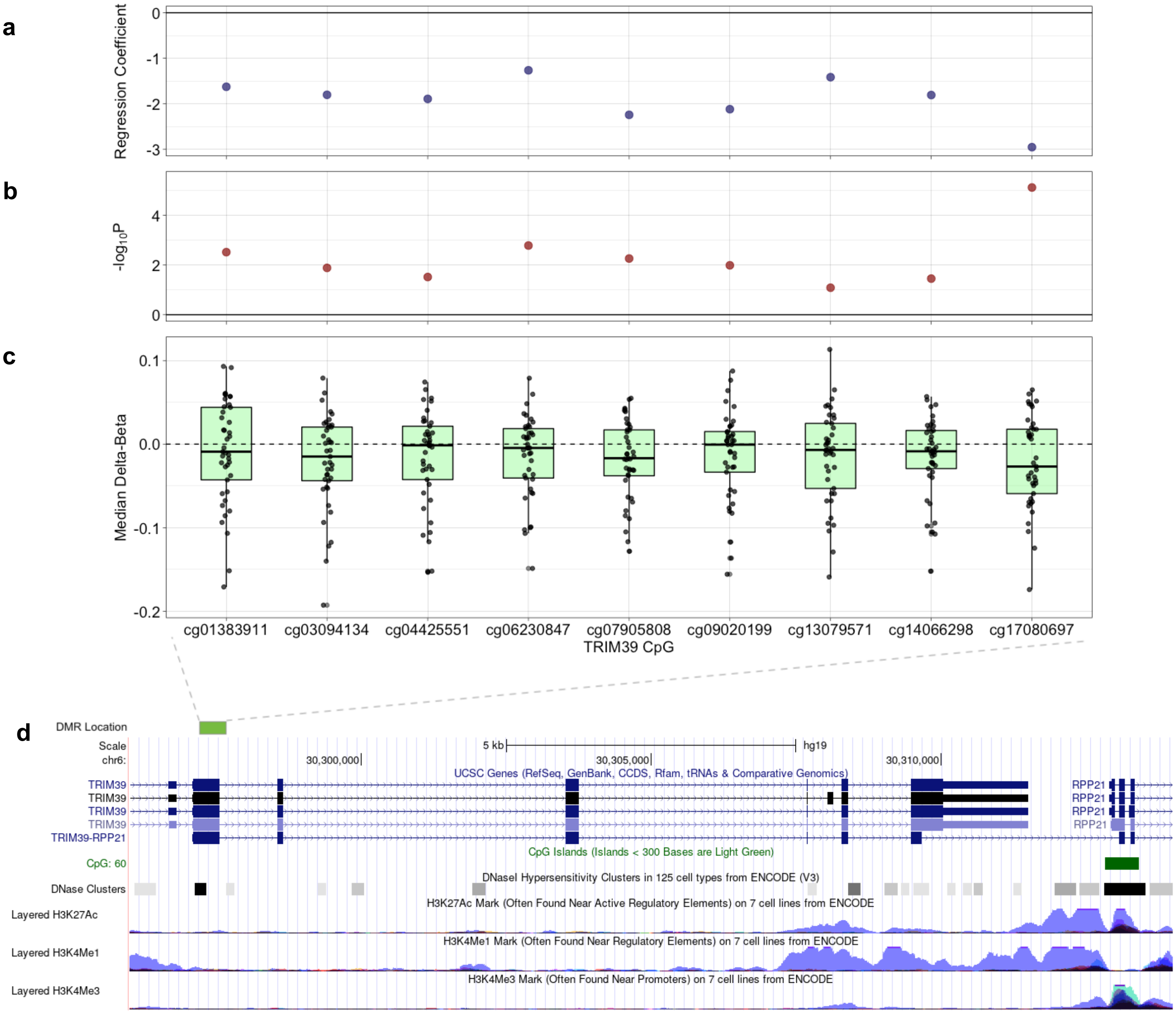

Supplementary Figure 5: Visualization of differentially methylated region associated with *TRIM39-RPP21*. Attributes of the 9 probes included in the significant differentially methylated region (DMR) at *TRIM39-RPP21* are shown, including **(a)** regression coefficients, **(b)** regression *P*-values and **(c)** boxplots of median delta-beta values (ALL-case DNA methylation beta value minus control beta) for n = 41 independent twin pairs. Regression coefficients and P-values represents results of conditional logistic regression evaluating the association between

ALL case status and DNA methylation at the listed CpGs controlling for sex, array chip, nucleated cell proportions and clustering by twin pair identity. **(d)** The corresponding *TRIM39* region in chromosome 6 as displayed in the UCSC Genome Browser (https://genome.ucsc.edu/) with the DMR position labeled in green. No CpG islands or regulatory elements from ENCODE (Layered H3K27Ac, H3K4Me1, H3L4Me3) are associated with the region, however a DNase sensitive site overlaps with three probes in the region. In boxplots, box represents interquartile range (IQR, first through third quartile) with centerline showing median value for all twin pairs, whiskers show minimum (first quartile minus 1.5*IQR) and maximum (third quartile plus 1.5*IQR) data range.

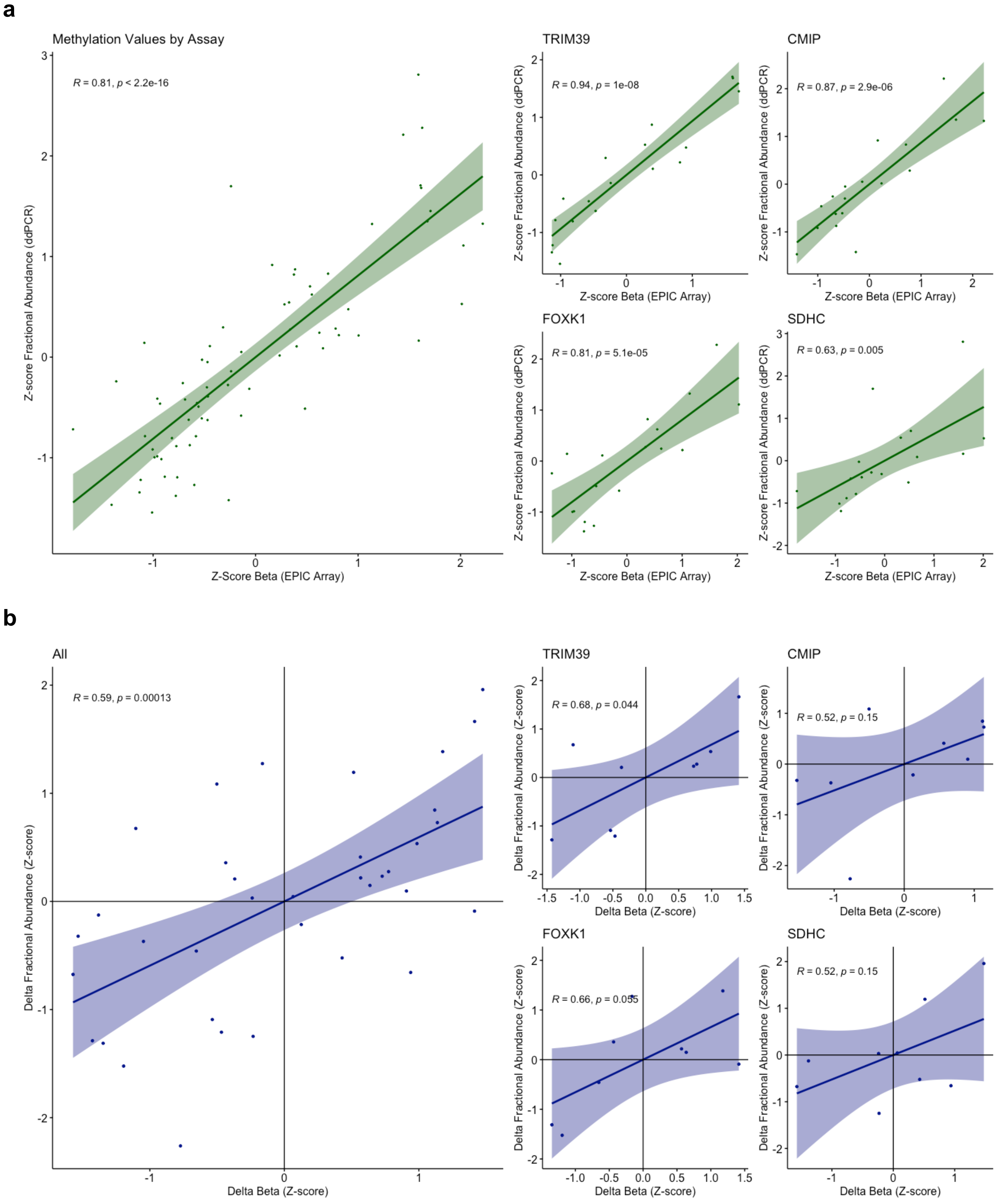

Supplementary Figure 6: **Validation of differentially methylated probes.** To validate a subset of significant differentially methylated probes identified from the EPIC array, DNA-

methylation specific droplet digital PCR (ddPCR) was conducted on twin pairs with adequate remaining genomic DNA (*n* = 9 pair) for four significant DMPs associated (note cg numbers here) with *FOXK1, TRIM39, CMIP, and SDHC*. DNA methylation level identified through ddPCR is represented by fractional abundance (FA), or the fraction of DNA methylation positive droplets divided by the total number of positive methylated and unmethylated droplets. **(a)** Direct comparison of FA and array beta values by subject. Left panel demonstrates combined data for all target sites and subjects assessed (*n* = 72), with Pearson *R* = 0.95. Target-site specific values (*n* = 18 each) are shown on the right panel, with *R* = 0.94 for *TRIM39, R* = 0.87 for *CMIP, R* = 0.81 for *FOXK1*, and *R* = 0.56 for *SHDC*. Overall and target-specific correlation are all significant (Two-sided Pearson correlation coefficient *P*-values ranging <2.2e-16 to *P* = 0.017). **(b)** Comparison of normalized (Z-score) site-specific delta-FA (case FA minus control FA) and delta-beta values. Overall results (*n* = 36 total target/pairs) are significantly correlated (two-sided Pearson correlation coefficient *R* = 0.56, *P* = 0.0034). Target-specific results show significant correlation for *TRIM39* (*R* = 0.68, *P* = 0.044) and borderline significance for *FOXK1* (*R* = 0.66, *P* = 0.055). Both *CMIP* and *SDHC* are positively correlated (*R* = 0.52 and 0.4, respectively) though not significant. A total of 26 of 36 targets have a consistent direction of delta DNA methylation difference (two-sided binomial test *P* = 0.01133). Plots show regression line with shaded area representing 95% confidence interval.

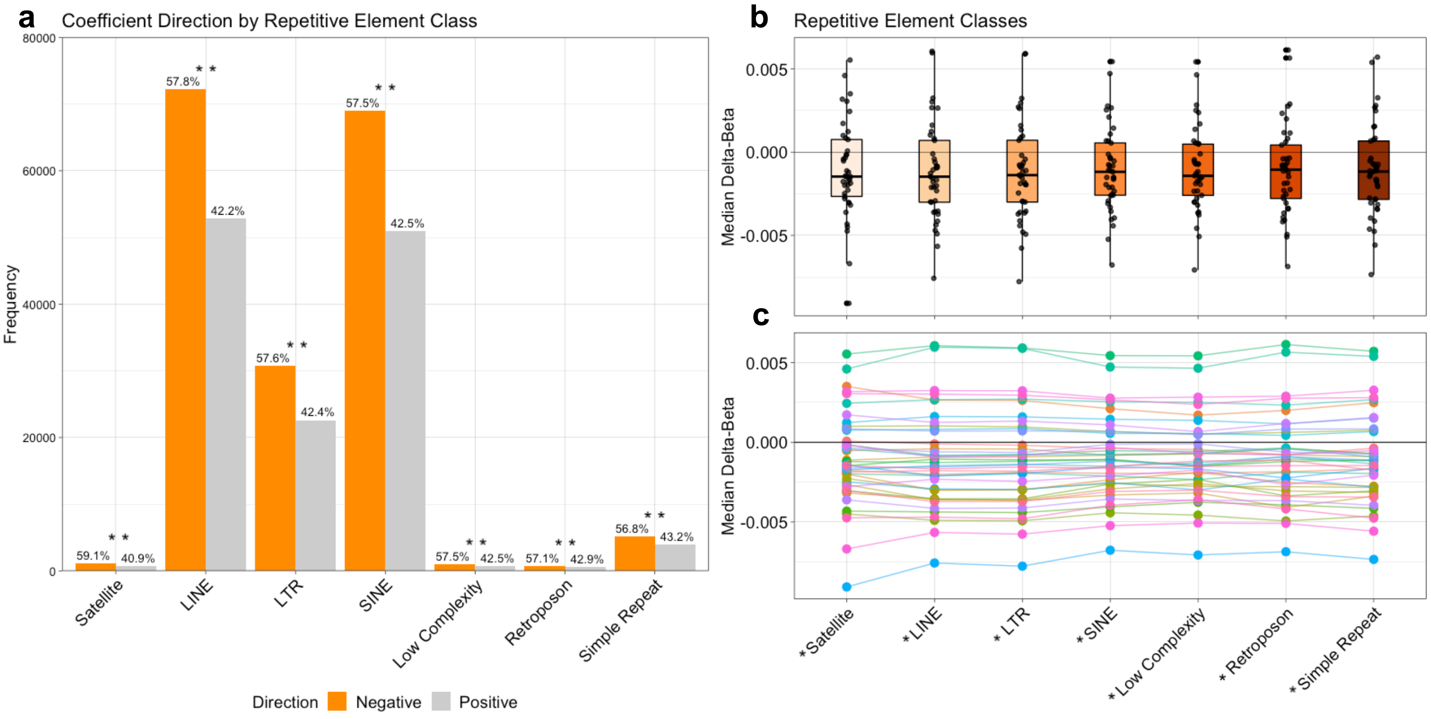

Supplementary Figure 7: Evaluation of DNA hypomethylation within repetitive elements. **(a)** Bar plot demonstrating frequency of positive and negative coefficients associated with probes overlapping repetitive element (RE) classes. Classes with at least 500 associated probes are shown. All repetitive element classes show a significant bias toward negative coefficients by two-sided binomial test. **(b)** Boxplot of median delta beta values in probes associated with RE classes for *n* = 41 independent twin pairs. Median values in 11 of 19 RE classes were significantly shifted toward DNA hypomethylation in cases (Wilcoxon rank-sum test), including the 8 classes represented on the plot. **(c)** Scatter plot of median delta beta values with lines connecting individual pair values across RE classes for *n* = 41 independent twin pairs. A consistent hypomethylation profile is evident across all RE classes for 30 of the 41 twin pairs. In boxplots, box represents interquartile range (IQR, first through third quartile) with centerline showing median value for all twin pairs, whiskers show minimum (first quartile minus 1.5*IQR) and maximum (third quartile plus 1.5*IQR) data range. *Two-sided Wilcoxon rank-sum test FDR<0.05. ***Two-sided binomial test FDR<0.001.

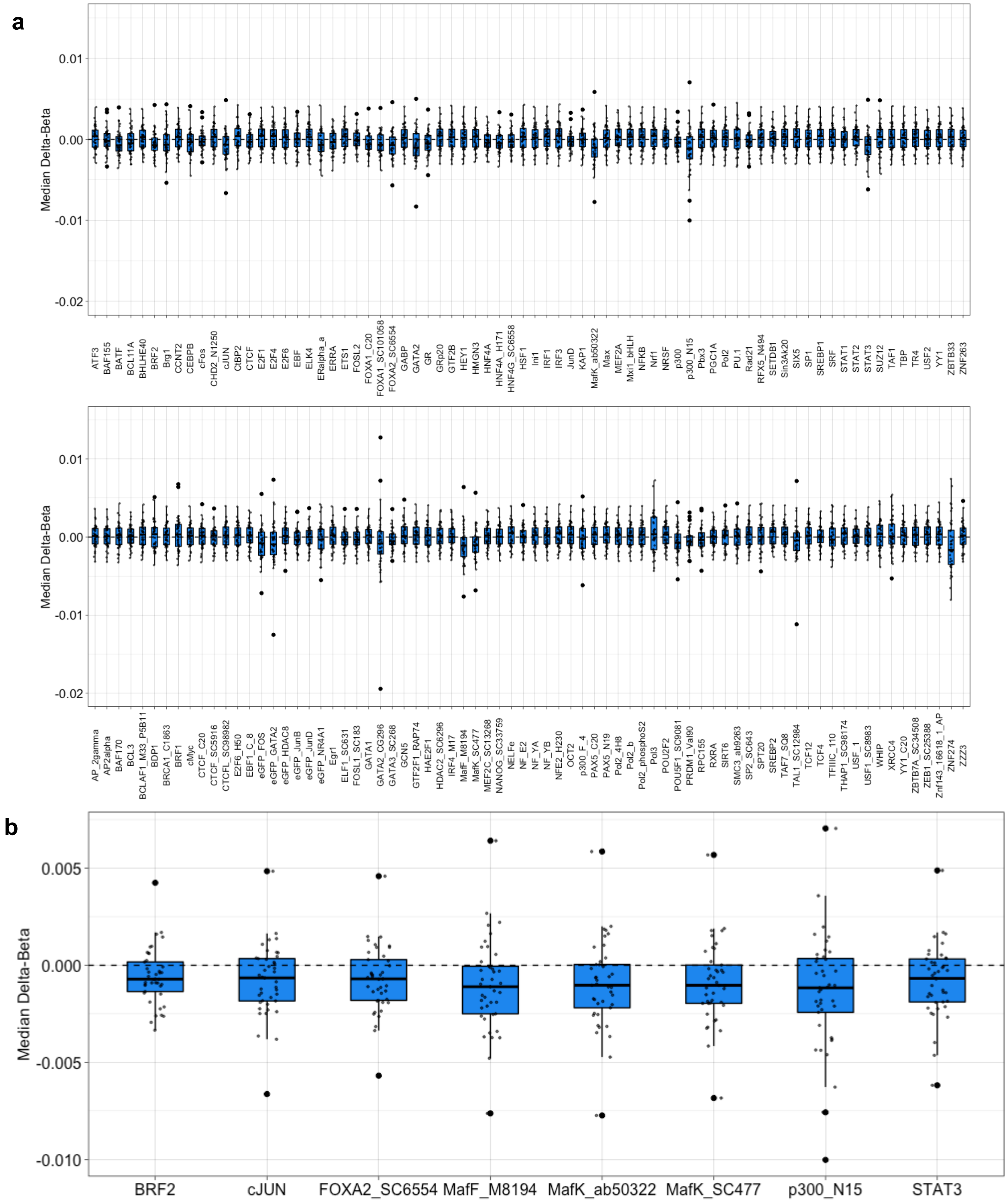

Supplementary Figure 8: Boxplot of median delta beta values for probes associated with transcription factor (TF)-motifs (*n* = 148). A plot of median delta beta values for all TF-motifs assessed is shown in **(a)**. 104 of 148 TF motifs has positive composite median values (across

all twin pairs), compared to 44 negative median values. While no TF-motif median delta beta values are significantly shifted (two-sided Wilcoxon signed-rank), 8 meet a more lenient threshold of FDR<0.1 **(b)**, all of which are demonstrate a tendency toward hypomethylation in ALL cases compared to matched siblings. In boxplots, box represents interquartile range (IQR, first through third quartile) with centerline showing median value for all twin pairs, whiskers show minimum (first quartile minus 1.5*IQR) and maximum (third quartile plus 1.5*IQR) data range.

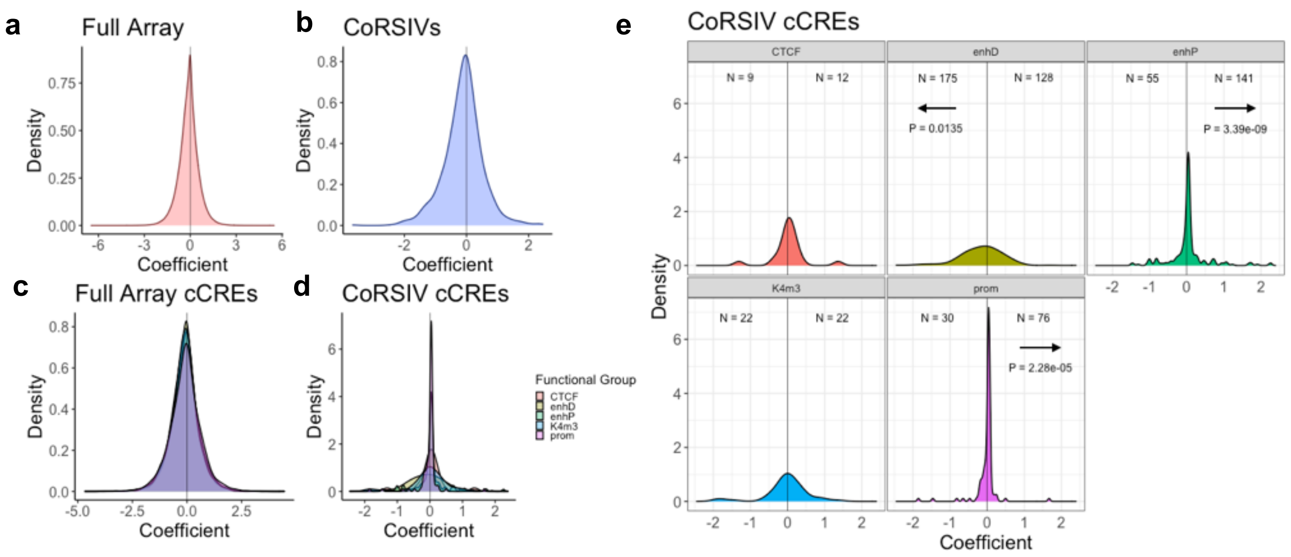

Supplementary Figure 9**: Negative coefficient bias across correlated regions of systemic interindividual variation (CoRSIV) and candidate cis-regulatory elements (cCRE). (a-b)** A total of 1,128 probes evaluated in conditional regression analysis overlapped with 756 distinct CoRSIVs across the genome, with a similar distribution of negative coefficients (55.9%) compared to the 710,010 overall array probes (57.7%). **(c)** A total of 88,233 probes from the full array overlapped with cCREs, with a uniform distribution across CTCF, distal enhancers (enhD), proximal enhancers enhP), K4m3 sites, and promoters (prom). **(d)** In contrast, the 670 probes in CoRSIVs overlapping cCREs were biased toward positive coefficients. **(e)** Of all cCREs assessed in CoRSIVs, only distal enhancers had a significant negative bias in coefficients. Significant (*P*<0.05) binomial test *P*-values are represented on plots.
