# Supplementary material for: DNA methylation at birth in monozygotic twins discordant for pediatric acute lymphoblastic leukemia
Source: Nat Commun. 2022 Oct 14;13:6077. doi: 10.1038/s41467-022-33677-z (PMC9568651; doi:10.1038/s41467-022-33677-z)
Supplement: Supplementary file 3 — Description of Additional Supplementary Files [file 41467_2022_33677_MOESM3_ESM.pdf]

Description of Additional Supplementary Files

File Name: Supplementary Data 1
Description: **Deconvolution analysis of *n* = 41 twin pairs included in analysis.** *P*-value represents results of a two-sided paired Wilcoxon test.

File Name: Supplementary Data 2
Description: **Recurrently variable CpGs in within-pair analysis.** Frequency represents number of pairs in which delta beta (case beta value minus control beta value) is equal to or exceeds 0.15, which represents an 15% absolute difference in beta value between individuals. Probes without gene names are intergenic and not associated with an identifiable nearby gene. SD = standard deviation.

File Name: Supplementary Data 3
Description: **Within-pair gene ontology analysis.** Gene ontology (GO) terms associated with 3,937 recurrently variable probes (absolute delta beta variation at least 15% and present in at least 2 twin pairs) from within-pair analysis are shown, generated from the gometh function of the missMethyl R-package which assesses for GO term enrichment using Wallenius' non-central hypergeometric test. N = number of genes associated with GO terms, DE = number of genes associated with each term for CpGs in analysis, P.DE = *P*-value for overrepresentation of term in CpGs in analysis, FDR = adjusted *P*-Value.

File Name: Supplementary Data 4
Description: **Within-pair KEGG pathway analysis.** KEGG-pathway terms associated with 3,937 recurrently variable probes (absolute delta beta variation at least 15% and present in at least 2 twin pairs) from within-pair analysis are shown, generated from the gometh function of the missMethyl R-package which assesses for KEGG pathway enrichment using Wallenius' non-central hypergeometric test. N = number of genes associated with KEGG terms, DE = number of genes associated with each term for CpGs in analysis, P.DE = *P*-value for overrepresentation of term in CpGs in analysis, FDR = adjusted *P*-Value.

File Name: Supplementary Data 5
Description: **Characteristics of array probes and conditional regression results.** A total of 710,010 CpG probes were analyzed using conditional logistic regression to assess the relationship between leukemia status and DNA methylation at each array CpG, controlling for sex, array plate, nucleated cell proportions, and clustering by twin pair identity. Mean values are displayed for each CpG by the overall cohort assessed in conditional regression (*n* = 74), and within cases and controls separately. Delta Beta values represent means of case minus control values. A total of 240 CpG probes reached a significance threshold of FDR<0.05. SD = Standard deviation. SE = Standard Error. FDR = False discovery rate. UCSC = University California Santa Cruz.

File Name: Supplementary Data 6
Description: **Gene ontology analysis of *n* = 240 significant CpGs in conditional regression analysis.** Gene ontology assessment conducted using the gometh function of the missMethyl R-package which assesses for GO term enrichment using Wallenius' non-central hypergeometric test. N = number of genes associated with GO terms, DE = number of genes associated with each term for CpGs in analysis, P.DE = *P*-value for overrepresentation of term in CpGs in analysis, FDR = adjusted *P*-Value.

File Name: Supplementary Data 7
Description: **KEGG pathway analysis of *n* = 240 significant CpGs in conditional regression analysis.** KEGG pathway analysis conducted using the gometh function of the missMethyl R-package which assesses for KEGG pathway enrichment using Wallenius' noncentral hypergeometric test. N = number of

genes associated with KEGG terms, DE = number of genes associated with each term for CpGs in analysis, P.DE = *P*-value for overrepresentation of term in CpGs in analysis, FDR = adjusted *P*-Value.

File Name: Supplementary Data 8
Description: **Probe level data for differentially methylated regions identified in comb-p analysis.** Probe characteristics including mean beta (across all subjects evaluated in regression analysis, $n = 37$) and standard deviation (SD), mean and SD for delta-beta values (case - control beta value), along with coefficient and raw *P*-value from conditional regression analysis.

File Name: Supplementary Data 9
Description: **Gene Ontology Analysis of $n = 10$ significant differentially methylated regions from comb-p analysis.** Gene ontology assessment conducted using the gometh function of the missMethyl R-package which assesses for GO term enrichment using Wallenius' non-central hypergeometric test. N = number of genes associated with GO terms, DE = number of genes associated with each term for CpGs in analysis, P.DE = *P*-value for overrepresentation of term in CpGs in analysis, FDR = adjusted *P*-Value.

File Name: Supplementary Data 10
Description: **KEGG pathway analysis of $n = 10$ significant differentially methylated regions from comb-p analysis.** KEGG pathway assessment conducted using the gometh function of the missMethyl R-package which assesses for KEGG pathway enrichment using Wallenius' non-central hypergeometric test. N = number of genes associated with KEGG terms, DE = number of genes associated with each term for CpGs in analysis, P.DE = *P*-value for overrepresentation of term in CpGs in analysis, FDR = adjusted *P*-Value.

File Name: Supplementary Data 11
Description: **Gene-target specific droplet digital PCR (ddPCR) results by twin pair.** A total of 9 twin pairs with sufficient sample were evaluated using DNA-methylation specific ddPCR at four CpGs targeting significant sites from conditional regression analysis in *CMIP*, *FOXK1*, *TRIM39*, and *SDHC*. Twin Pair ID denotes twin relationships (designation has been randomly assigned for supplementary tables). Total accepted droplets represent the total number of droplets analyzed. Positive and negative droplets are shown for methylated and unmethylated probes. Fractional abundance represents the proportion of positive methylated droplets to all positive (methylated and unmethylated) droplets, presented as percentage methylated.

File Name: Supplementary Data 12
Description: **Comparison of droplet digital PCR (ddPCR) and array-based DNA methylation results in four significant CpG targets.** A total of 9 twin pairs with sufficient sample were evaluated using DNA-methylation specific ddPCR at four CpGs targeting significant sites from conditional regression analysis in *CMIP*, *FOXK1*, *TRIM39*, and *SDHC*. Twin Pair ID denotes twin relationships (designation has been randomly assigned for supplemental tables). A comparison between methylation levels detected by methylationspecific ddPCR (fractional abundance) and DNA methylation array (beta) is shown by twin pairing. Delta (case - control) values were normalized for comparison between methods. SD = standard deviation.

File Name: Supplementary Data 13
Description: **Global DNA methylation comparison by case status.** Comparison of median global DNA methylation content (representing the average beta DNA methylation value across all probes on the array) by case status in 41 discordant twin pairs. Pairs are arranged in rows without identifiers. Cases demonstrate significant hypomethylation compared to control twins (two-sided paired Wilcoxon test $P = 0.048$). IQR = interquartile range.

File Name: Supplementary Data 14
Description: **Coefficient distribution and median delta beta values by repetitive element class.** For assessment of coefficient directions, *P*-value represents twosided binomial test, with false discovery rate (FDR) correction listed to account for multiple comparisons. For assessment of delta beta values, *P*-value represents results of a two-sided Wilcoxon rank-sum test, with FDR listed to account for multiple comparisons.

File Name: Supplementary Data 15
Description: **Transcription factor enrichment in significant probes in conditional regression analysis.** Enrichment column refers to whether transcription factor binding motif overlaps with a greater proportion of significant probes compared to overlaps in the full array (enriched) or a lesser proportion (depleted). Statistical columns represent results of a two-sided Fisher's exact test for each transcription factor binding site motif. OR = odds ratio.

File Name: Supplementary Data 16
Description: **Coefficient distribution and median delta beta values by transcription factor motif.** For assessment of coefficient directions, *P*-value represents twosided binomial test, with false discovery rate (FDR) correction listed to account for multiple comparisons For assessment of delta beta values, *P*-value represents results of a two-sided Wilcoxon rank-sum test, with FDR listed to account for multiple comparisons. IQR = interquartile range.

File Name: Supplementary Data 17
Description: **Coefficient distribution by candidate cis-regulatory element (cCRE) and by cCRE/correlated regions of systemic interindividual variation (CoRSIV) overlapping sites.** *P*-value represents results of a two-sided binomial test, with false discovery rate (FDR) listed to account for multiple comparisons. CI = confidence interval.

File Name: Supplementary Data 18
Description: **Probe and primer design for droplet digital PCR (ddPCR) analysis.** Shown are forward and reverse primers designed using MethPrimer for methylationspecific DNA binding, along with probes designed to target the listed CpG site in the methylated and unmethylated state. The targeted CpG site is bolded. bp = base pairs. C = Celsius
