# Supplementary material for: DNA methylation at birth in monozygotic twins discordant for pediatric acute lymphoblastic leukemia
Source: Nat Commun. 2022 Oct 14;13:6077. doi: 10.1038/s41467-022-33677-z (PMC9568651; doi:10.1038/s41467-022-33677-z)
Supplement: Supplementary file 6 — Reporting Summary [file 41467_2022_33677_MOESM6_ESM.pdf]

nature portfolio

Corresponding author(s): Joseph L Wiemels

Last updated by author(s): Sep 5, 2022

Reporting Summary

Nature Portfolio wishes to improve the reproducibility of the work that we publish. This form provides structure for consistency and transparency in reporting. For further information on Nature Portfolio policies, see our Editorial Policies and the Editorial Policy Checklist.

Statistics

For all statistical analyses, confirm that the following items are present in the figure legend, table legend, main text, or Methods section.

n/a	Confirmed	
☐	☒	The exact sample size (*n*) for each experimental group/condition, given as a discrete number and unit of measurement
☒	☐	A statement on whether measurements were taken from distinct samples or whether the same sample was measured repeatedly
☐	☒	The statistical test(s) used AND whether they are one- or two-sided<br>*Only common tests should be described solely by name; describe more complex techniques in the Methods section.*
☐	☒	A description of all covariates tested
☐	☒	A description of any assumptions or corrections, such as tests of normality and adjustment for multiple comparisons
☐	☒	A full description of the statistical parameters including central tendency (e.g. means) or other basic estimates (e.g. regression coefficient) AND variation (e.g. standard deviation) or associated estimates of uncertainty (e.g. confidence intervals)
☐	☒	For null hypothesis testing, the test statistic (e.g. *F*, *t*, *r*) with confidence intervals, effect sizes, degrees of freedom and *P* value noted<br>*Give P values as exact values whenever suitable.*
☒	☐	For Bayesian analysis, information on the choice of priors and Markov chain Monte Carlo settings
☒	☐	For hierarchical and complex designs, identification of the appropriate level for tests and full reporting of outcomes
☐	☒	Estimates of effect sizes (e.g. Cohen's *d*, Pearson's *r*), indicating how they were calculated

Our web collection on statistics for biologists contains articles on many of the points above.

Software and code

Policy information about availability of computer code

Data collection	No software was used in data collection.
Data analysis	Analysis of PMDA array genotyping data, including identity by descent determination, was conducted using PLINK (version 1.90). Analysis of EPIC array DNA methylation data was conduced in R (version 4.0.0). Open source R packages used for data analysis in this study include the following: "SeSAMe" was used for data normalization and quality control measures for raw DNA methylation array IDAT files, "impute" was used for imputation of missing values, "Rtsne" was used for tSNE analysis, “FlowSorted.Blood.EPIC” was used for nucleated cell deconvolution analysis, "missMethyl" was used for gene set enrichment analysis, "survivor" was used for conditional regression analysis, “IlluminaHumanMethylationEPICanno.ilm10b4.hg19” was used for EPIC array annotation. Determination of differentially methylated regions was conducted using “comb-P" in Python (version 3.7.6). Droplet digital PCR data was analyzed using Bio-Rad’s QuantaSoft Analysis Pro Software (version 1.0596). Code used for the conditional logistic regression analysis described in this manuscript is available for download from the Harvard Dataverse (https://doi.org/10.7910/DVN/NT1WAX).

For manuscripts utilizing custom algorithms or software that are central to the research but not yet described in published literature, software must be made available to editors and reviewers. We strongly encourage code deposition in a community repository (e.g. GitHub). See the Nature Portfolio guidelines for submitting code & software for further information.

Data

Policy information about availability of data

All manuscripts must include a data availability statement. This statement should provide the following information, where applicable:

- Accession codes, unique identifiers, or web links for publicly available datasets
- A description of any restrictions on data availability
- For clinical datasets or third party data, please ensure that the statement adheres to our policy

This study used biospecimens from the California Biobank Program. Per California Health and Safety Code Sections 124980(j), 124991(b), (g), (h), and 103850 (a) and (d), which protects the confidentiality of data obtained from biospecimens, we are respectfully unable to share raw, individual level genomic and genome-wide DNA methylation data reported in this study, which are property of the State of California. Should we be contacted regarding individual level data contributing to the findings reported in this study, inquiries will be directed to the California Department of Public Health Institutional Review Board to establish an approved protocol to utilize the data, which cannot otherwise be shared peer-to-peer. The State of California has provided guidance on data sharing per the following statement: "California has determined that researchers requesting the use of California Biobank biospecimens for their studies will need to seek an exemption from NIH or other granting or funder requirements regarding the uploading of study results into an external bank or repository (including into the NIH dbGaP or other bank or repository). This applies to any uploading of genomic data and/or sharing of these biospecimens or individual data derived from these biospecimens. Such activities have been determined to violate the statutory scheme at California Health and Safety Code Section 124980 (j), 124991 (b), (g), (h) and 103850 (a) and (d), which protect the confidential nature of biospecimens and individual data derived from biospecimens. All investigators seeking to use California specimens for projects or grant related activities that require or seek such sharing (at the NIH or elsewhere) must seek an exemption from genomic data sharing requirements. If such an exemption is not secured, samples will not be released to an investigator. Investigators may agree to share aggregate data on SNP frequency and their associated P-values with other investigators and may upload such frequencies into repositories including the NIH dbGaP repository providing a) the denominator from which the data is derived includes no fewer than 20,000 individuals; b) no cell count is for < 5 individuals; and c) no correlations or linkage probabilities between SNPs are provided." Datasets evaluated in this manuscript include the California Cancer Registry and California Birth Records Master Statistical File. Source data for Figures 1, 2, and Supplementary Figures 1 and 2 are provided as a Source data file with this paper. Conditional logistic regression results are presented in Source data file (under Figure 1a) and in annotated form as Supplementary Data 5. The delta beta values (leukemia case DNA methylation beta value minus control beta value) generated in this study have been deposited in the Harvard Dataverse (https://doi.org/10.7910/DVN/NT1WAX).

Field-specific reporting

Please select the one below that is the best fit for your research. If you are not sure, read the appropriate sections before making your selection.

☒ Life sciences ☐ Behavioural & social sciences ☐ Ecological, evolutionary & environmental sciences

For a reference copy of the document with all sections, see nature.com/documents/nr-reporting-summary-flat.pdf

Life sciences study design

All studies must disclose on these points even when the disclosure is negative.

Sample size	The total sample size assessed in this study is n = 86 subjects (43 twin pairs). Of these, n = 82 individuals (41 twin pairs) met threshold outline within the methods section for evaluation of DNA methylation array data and were included in our data analysis. We did not perform a sample size calculation; given the rarity of our sample set, we used all available subjects fitting requirements for inclusion in the study from available registry data.
Data exclusions	We excluded n = 4 individuals (2 twin pairs) included in the study from our data analysis, including the within-pair analysis and conditional regression analysis. These individuals were excluded due to failure to meet quality control thresholds outlined within the methods section of our paper for use of array data. Specifically, 'detect-P' values were significantly elevated in these subjects, precluding their use in analysis as outlined in the manuscript.
Replication	Due to the limited amounts of genomic DNA available for analysis, and the prohibitive cost of conducting the DNA methylation array described in the manuscript, replication of array data was not conducted for any of the subjects in this study. We conducted a separate analysis using a different methodology (DNA-methylation specific droplet digital PCR) as a means of validation of the array results for a subset of significant findings.
Randomization	While the retrospective design of this study does not facilitate randomization of experimental treatments, we utilized block randomization during bisulfite treatment of subject DNA. We additionally randomized subjects to separate BeadChips for array analysis, ensuring twin siblings were evaluated on separate chips.
Blinding	Blind identification codes (known to the laboratory manager and principle investigator) were utilized throughout the study to both prevent identification of subjects and limit bias. Given the twin nature of the dataset, identification of twin pairings was maintained throughout the study.

Reporting for specific materials, systems and methods

We require information from authors about some types of materials, experimental systems and methods used in many studies. Here, indicate whether each material, system or method listed is relevant to your study. If you are not sure if a list item applies to your research, read the appropriate section before selecting a response.

Materials & experimental systems

n/a	Involved in the study
☒	☐ Antibodies
☒	☐ Eukaryotic cell lines
☒	☐ Palaeontology and archaeology
☒	☐ Animals and other organisms
☐	☒ Human research participants
☒	☐ Clinical data
☒	☐ Dual use research of concern

Methods

n/a	Involved in the study
☒	☐ ChIP-seq
☒	☐ Flow cytometry
☒	☐ MRI-based neuroimaging

Human research participants

Policy information about studies involving human research participants

Population characteristics

This study involved monozygotic (identical) twins identified through combined California Cancer Registry and California birth records data from 1988-2015 in which one individual of a twin pair developed pediatric (0-23 years) acute lymphoblastic leukemia while the remaining twin sibling did not. This included a total of 43 twin pairs with available archived dried neonatal blood spots for analysis who were confirmed to be monozygotic through SNP-array assessment. Of these 43 twin pairs, 27 were female and 16 were male. Age of diagnosis in the case twin ranged from 0 to 23 years, with a median age of 5. The lineage of ALL diagnosis was noted as B-cell in 32 cases, T-cell in 4 cases, and unknown in 7 cases.

Recruitment

Subjects were not actively recruited for inclusion in the study. Rather, retrospective assessment of available registry data from the California Cancer Registry and birth records were used to identify subjects.

Ethics oversight

The procedures for obtaining archived dried blood spots (DBS) from the California Department of Public Health (CDPH) has already passed human subjects approval at the State of California institutional review board (IRB) with reliances at other institutions, including IRB approval from the University of Southern California (USC). The CDPH Genetic Diseases Screening Branch obtains DBS from all neonates born within the state with the purpose the Newborn Screening Program (NBS). Following use in the NBS, remaining samples are archived under the California Biobank Program and are made available for use in appropriate scientific investigations under an “opt-out” mechanism per Section 6505 of Title 17 of the California Administrative Code. Subjects and parents of subjects are thus not required to opt in to selected appropriate scientific investigations. Samples are stored and can be accessed for research purposes with approval from the California Health and Human Services Agency’s (CHHSA) Committee on the Protection of Human Subjects (CPHS). Approval from the CPHS for samples to be used in this proposal has been obtained under protocol #17-04-2958.

Note that full information on the approval of the study protocol must also be provided in the manuscript.
